# Increased inflammatory markers in adult patients born with an atrial septal defect

**DOI:** 10.3389/fcvm.2022.925314

**Published:** 2022-08-01

**Authors:** Anne-Sif Lund Schram, Anna Sellmer, Camilla Nyboe, Martin Sillesen, Vibeke Elisabeth Hjortdal

**Affiliations:** ^1^Department of Cardiothoracic Surgery, Copenhagen University Hospital, Rigshospitalet, Copenhagen, Denmark; ^2^Department of Anesthesiology, Aarhus University Hospital, Aarhus, Denmark; ^3^Department of Surgical Gastroenterology and Transplantation, Copenhagen University Hospital, Rigshospitalet, Copenhagen, Denmark; ^4^Center for Surgical Translational and Artificial Intelligence Research (CSTAR), Copenhagen University Hospital, Rigshospitalet, Copenhagen, Denmark; ^5^Department of Clinical Medicine, Aarhus University, Aarhus, Denmark

**Keywords:** congenital heart disease, atrial septal defect, inflammation, cytokines, inflammatory activity

## Abstract

Patients with atrial septal defect (ASD) have higher mortality and higher risk of atrial fibrillation, heart failure, pneumonia, and stroke than the general population even if the ASD closes spontaneously in childhood. The reason for the long-term complications remains unknown. Since many of the complications can be linked up with alterations in inflammatory response, we speculate that inflammation may contribute to the association between ASD and morbidity and mortality. We investigated inflammatory activity in adults with an ASD compared with controls. We included 126 adults with an unrepaired ASD. A group of healthy controls were recruited as comparison group (*n* = 23). Serum samples were analyzed for 92 inflammation-related protein biomarkers using a proximity extension assay. A pathway enrichment analysis was performed using Reactome database. Out of 92 biomarkers, 73 were eligible for data analysis. Increased levels of 14 (19%) biomarkers were found in patients with open ASD and 24 (33%) biomarkers in patients with spontaneously closed defects compared with controls (*p* < 0.05). Multiple inflammatory pathways showed stronger enrichment in both patient groups when compared with controls. In conclusion, inflammatory activity is altered in adult patients with an unrepaired ASD compared with healthy controls. The increased inflammatory burden of patients with an unrepaired ASD may contribute to the development of morbidities.

## Introduction

Recent discoveries have led to the understanding, that an atrial septal defect (ASD) is not as benign as previously thought. We have demonstrated that patients with an ASD have a higher long-term mortality compared with the background population, both in patients with and without prior closure of the defect (1), and the most frequent cause of death being non-ischemic cardiac disease (1, 2). Patients with an ASD have an increased long-term risk of atrial fibrillation, stroke (3) and pneumonia (4), as well as a higher incidence of unemployment and psychiatric disorders compared with background population (5–7).

There is little evidence of the underlying cause and the current opinion is that a combination of genetic, epigenetic and environmental factors is implicated (8, 9).

We have documented an increased burden of pathogenic gene variants in patients with a small ASD (8). The pathway between the gene variation and the phenotypic presentation of morbidity in these patients is unknown. It is our speculation that gene variants may be responsible for an altered immunological response in patients with congenital heart defects and accumulating evidence indicates inflammatory alterations in children and adults with congenital heart disease (CHD) (9, 10). It is suggested that the immune system is involved in the complications of CHD (9, 10). Inflammatory markers, such as high-sensitive c-reactive protein have been found to be an independent predictor of cardiovascular adverse events and mortality in adults with CHD, including patients with simple ventricular or atrial shunts (11). Inflammation is contributing to pathological myocardial remodeling, fibrosis and heart failure (12–14), and is considered an independent risk factor for atrial fibrillation and a mediator of a prothrombotic state (15, 16). Moreover, inflammation plays a role in pulmonary hypertension by inducing vascular remodeling and endothelial proliferation (17), and may also increase the susceptibility of developing pneumonia (18).

Patients diagnosed with a hemodynamically insignificant ASD are not offered closure. These unrepaired ASDs have surprisingly been found to be equally problematic as repaired ASDs (1–7).

Despite that the association between ASD and increased morbidity and mortality is well-described, the understanding of inflammation in relation to the complications is limited. We hypothesize that perturbations of the inflammatory systems contribute to the pathophysiological mechanisms. In this exploratory study we aimed to clarify if the pattern of a broad range of inflammation-related biomarkers is altered and if the inflammatory biological processes are overrepresented in patients with an unrepaired ASD compared to controls.

## Materials and methods

### Study population and design

Patients were included from a nationwide cohort comprising all patients diagnosed with an unrepaired ASD from 1977 to 2013 (1). Patients were identified through the Danish National Patient Registry (19), and the diagnosis: ASD was validated manually. A detailed description of the method is described in previous studies (3, 4). From the nationwide cohort, we excluded patients with persistent foramen ovale, pulmonary arterial hypertension, Eisenmenger syndrome, previous surgical or percutaneous closure of the defect, CHD (except patent ductus arteriosus), severe mental or severe psychiatric disorder to a degree where the patient was unable to cooperate and death.

Enrollment in the present study was conducted between December 2015 and November 2017. Patients between 18 and 65 years were invited for an extensive examination including a blood sample and an echocardiography, as described previously (2). For comparison, healthy control subjects were recruited through announcements in the local area and through an official website.

### Blood samples and protein analysis

Blood samples were obtained from a cubital vein in serum tubes (BD Vacutainer^®^, BD Plymouth, United Kingdom) and centrifuged for 20 min at 4°C and 4,000 rpm. Serum was collected and preserved at −80°C until further analysis.

A multiplex immunoassay biomarker panel (Olink Proteomics, Uppsala, Sweden) based on a proximity extension assay technique was used to analyze 92 established and exploratory inflammatory related biomarkers (Supplementary Table 1). Analyses were performed using BioMark™ HD, Fluidigm^®^ at BioXpedia A/S, Aarhus, Denmark. The proximity extension assay technique has previously been thoroughly described (20, 21). In brief, 1 μL of serum was incubated with 92 antibody pairs labeled with DNA oligonucleotides. The oligonucleotide-labeled antibody pairs were coupled to their respective target protein. When in proximity of corresponding antibody-probes, the oligonucleotides hybridized, and an extension reaction formed a unique target sequence. Subsequently, the target sequence was quantified using standard real-time quantitative polymerase chain reaction. The serum samples were distributed randomly and analyzed using two fluidigm plates.

External and internal controls were included in the proximity extension assay to monitor assay performance and adjust for intra- and inter-run variation. To each sample, 4 internal controls were added ensuring a strict quality control of assay performance in each step, i.e., antibody binding, extension and detection step. Negative controls were used to calculate assay specific limit of detection and define background signal. Interplate positive controls were used to calculate normalized protein expression (20). Normalization between plates was performed using intensity normalization. The proximity extension assay readout was normalized protein expression, which is an arbitrary unit on log2 scale where a high normalized protein expression-value equals a high protein concentration. Proteins with > 5% of normalized protein expression-values below limit of detection were excluded.

### Echocardiography

A standardized transthoracic echocardiography protocol was used to examine the right heart chambers and to determine if defects were open or spontaneously closed since the time of diagnoses. Moreover, this examination was used to sub-group patients with an open ASD into (1) hemodynamically significant open ASD. These were deemed the largest ASDs. (2) hemodynamically insignificant ASD. One highly trained echo-technician performed the examination using a GE Vivid E9 (GE Healthcare, Horten, Norway). A cardiologist specialized in CHD reviewed all images.

### Questionnaire

Participants completed a questionnaire regarding weight, height and smoking habits.

### Statistical analyses

Demographic data was evaluated by Mann-Whitney test for non-normally distributed continuous data and were compared by Fischer’s exact tests for categorical data. Demographic variables are presented as median and interquartile range or as number and percentages.

Results of biomarkers were compared using a linear regression model and are presented as crude and as adjusted for gender (categorical), age (continuous), smoking (categorical), and body mass index (continuous). Adjustments were also made for the presence of an estimated large ASD in the group of patients with an open ASD. Normalized protein expression-values for biomarker levels were presented as mean with standard deviation. *P*-values were *post hoc* corrected by the false detection rate, as proposed by Fabregat et al. (22). A false detection rate < 0.05 was considered statistically significant. Calculations were performed using Stata 15 (StataCorp LP, Texas, United States) and the “r” software package (23).

A pathway enrichment analysis was performed on biomarkers with significant differences between patient groups and controls using Reactome (24). The overrepresentation analysis is a hypergeometric distribution test, determining whether certain Reactome pathways of human biological processes are enriched in the submitted data. The test generates a probability score, which is corrected for false detection rate using the Benjamini-Hochberg method (22).

### Ethics

The study was approved by The Danish Data Protection Agency (jr.nr. 1-16-02-633-15), The Regional Committee on Biomedical Research Ethics of the Central Denmark Region (j.nr. M-2015-197-15) and The National Board of Health (j. nr. 7-604-04-2/193/KWH). The study was conducted in accordance with the ethical guidelines of the 1975 Declaration of Helsinki. Written and oral information were given, and all participants gave their informed consent prior to inclusion.

## Results

### Patient characteristics

**FIGURE 1:**
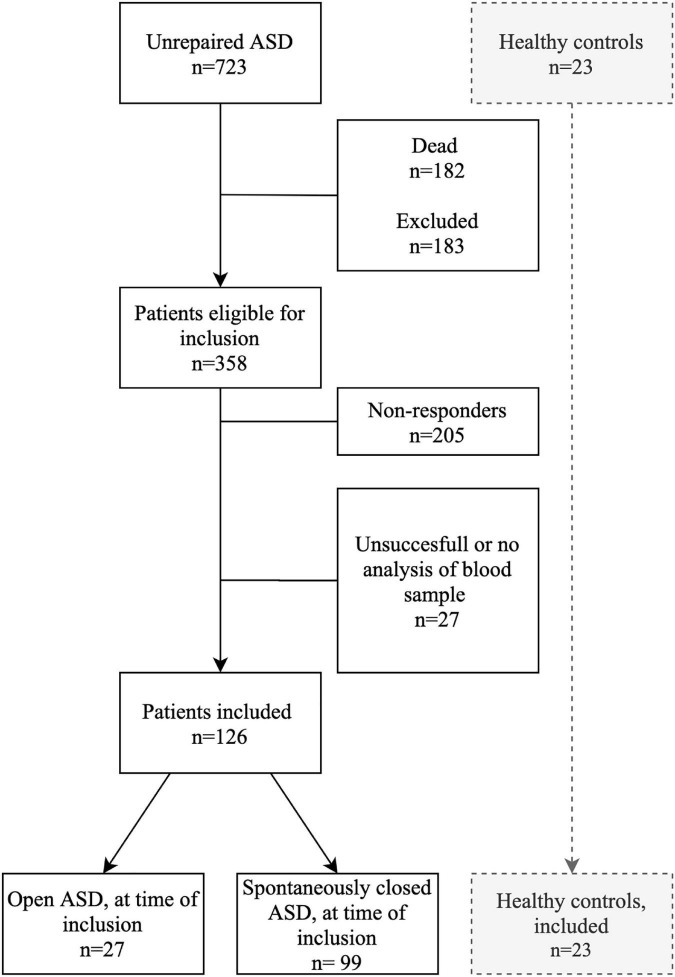
Flowchart of inclusion of patients with an unrepaired atrial septal defect. ASD, Atrial septal defect.

The validated Danish cohort of patients with ASD consists of 723 patients with an unrepaired ASD. The ASDs were deemed hemodynamically insignificant at the time of diagnosis and hence left unoperated. Of these, 182 patients had died since time of diagnosis. A further 183 patients were excluded due to: age > 65 (*n* = 88), emigration (*n* = 24), Downs syndrome (*n* = 12), psychiatric disorder (*n* = 34), repaired ASD since primary inclusion (*n* = 17), other CHD (*n* = 7), and Eisenmenger syndrome (*n* = 1). We invited 358 patients, out of which 153 patients participated. Further, two patients were unable to cooperate in the blood sample procedure, and two patients were excluded due to unacceptable technical variations in serum analysis, thus failing the quality control. Echocardiography disclosed that 27 were still open and the remaining had closed spontaneously. Restrained by a finite number of wells in the fluidigm plates, we chose to analyze blood samples from the 27 patients with an open ASD, 99 randomly chosen patients with a spontaneously closed ASD. Hence, a total of 126 ASD patients were enrolled in the study. Further, 23 healthy controls were included (Figure 1).

Characteristics of study subjects are outlined in Table 1. Patients with a spontaneously closed ASD were younger than patients with an open ASD and the controls. No other significant differences were found between patient groups and controls.

**TABLE 1:** Characteristics of 126 patients with an open atrial septal defect and 23 controls based on a nationwide cohort study in Denmark.

	Open ASD *n* = 27	Spontaneously closed ASD *n* = 99	Controls *n* = 23
Females, *n* (%)	17 (63)	59 (60)	14 (61)
Age, years	33 (26–41)	24 (22–35)*^†^	30 (26–41)
BMI, kg/m^2^	23.7 (20.4–29.0)	24.2 (21.9–28.1)	23.1 (22.0–24.2)
Weight, kg	72 (57–88)	72.5 (59–85)	67 (62–78)
Height, cm	170 (167–179)	170 (162–180)	172 (168–177)
Smoking, *n* (%)	8 (29.63)	25 (25.25)	3 (13.04)
Atrial fibrillation, *n* (%)	0 (0)	2 (2)	–
LV ejection fraction, %	62 (60–65)	60 (60–65)	–
RV FAC %	52 (50–55)	52 (50–56)	–
TR, mmHg	16 (11–19)	15 (10–19)	–
Pulmonary hypertension, *n*	0 (0)	0 (0)	–
Large ASD^‡^	11 (41)	–	–

Values are number (percentage) or median (interquartile range). 16 patients had no TR. ASD, Atrial septal defect; BMI, body mass index; LV, left ventricle; RV, right ventricle; FAC, fractional area change; TR, tricuspid valve regurgitant gradient. ^‡^Patients with right ventricular dilatation or pulmonary artery dilation. *p-value < 0.05 vs. controls, ^†^*p*-value < 0.05 vs. open ASD.

Right ventricular dilatation was seen in 10 patients with an open ASD, and one patient had pulmonary artery dilation. Closure of the defect was recommended in these patients as they were categorized as large or hemodynamically significant defects. No patients had heart failure or pulmonary hypertension defined as a mean pulmonary artery pressure above 25 mmHg estimated from tricuspid regurgitation peak velocity.

### Protein expression

A total of 19 biomarkers had > 5% of normalized protein expression-values below limit of detection and were excluded (listed in Supplementary Table 2), leaving 73 biomarkers eligible for further data analysis. Results of the 29 biomarkers with significant differences between patients with an ASD and controls are displayed in Table 2, whereas results of the 44 non-significant proteins are listed in Supplementary Table 3.

**TABLE 2:** Twenty nine inflammatory markers with significant different levels between patients with an atrial septal defect and controls.

Biomarker	Open ASD (*n* = 27)	Spontaneously closed ASD (*n* = 99)	Controls (*n* = 23)	Open ASD vs. controls		Spont. closed ASD vs. controls	
		
				Unadjusted *p*-value	Adjusted *p*-value	Unadjusted *p-*value	Adjusted *p*-value
AXIN1	3.06 ± 0.57	2.91 ± 0.51	1.71 ± 0.42	<0.01	<0.01	<0.01	<0.01
CASP8	0.92 ± 0.36	0.87 ± 0.33	0.57 ± 0.33	<0.01	0.06	<0.01	<0.01
CCL3	5.25 ± 0.55	5.12 ± 0.98	4.63 ± 0.62	<0.01	<0.01	0.06	0.18
CCL4	7.33 ± 0.52	7.28 ± 0.60	6.70 ± 0.78	<0.01	0.05	<0.01	<0.01
CCL11	7.83 ± 0.43	7.72 ± 0.46	7.48 ± 0.72	0.10	0.08	0.11	0.02
CCL23	9.82 ± 0.46	9.78 ± 0.38	10.00 ± 0.47	0.30	0.29	0.05	0.04
CD40	9.77 ± 0.34	9.74 ± 0.29	9.26 ± 0.28	<0.01	<0.01	<0.01	<0.01
CXCL1	8.62 ± 0.47	8.62 ± 0.51	8.10 ± 0.90	0.04	0.10	<0.01	0.02
CXCL6	8.78 ± 0.58	8.79 ± 0.55	7.83 ± 0.89	<0.01	<0.01	<0.01	<0.01
CXCL11	8.13 ± 0.63	8.08 ± 0.78	7.28 ± 0.98	<0.01	0.02	<0.01	<0.01
DNER	7.95 ± 0.27	8.07 ± 0.22	8.12 ± 0.21	0.05	0.03	0.51	0.91
GDNF	1.19 ± 0.45	1.19 ± 0.58	1.44 ± 0.41	0.10	0.20	0.12	0.04
HGF	8.53 ± 0.36	8.58 ± 0.39	8.13 ± 0.57	0.01	0.17	<0.01	<0.01
IL12β	4.37 ± 0.64	4.29 ± 0.61	4.07 ± 0.52	0.15	0.02	0.21	0.73
MCP1	10.64 ± 0.43	10.73 ± 0.43	10.28 ± 0.69	0.08	0.10	<0.01	<0.01
MCP2	8.43 ± 0.47	8.51 ± 0.68	7.59 ± 0.86	<0.01	0.01	<0.01	<0.01
MCP3	1.86 ± 0.64	1.84 ± 0.61	1.38 ± 0.58	0.03	0.10	<0.01	0.04
MCP4	4.87 ± 0.59	4.83 ± 0.59	3.91 ± 1.03	<0.01	0.02	<0.01	<0.01
MMP1	13.75 ± 0.77	13.58 ± 0.83	12.68 ± 1.26	<0.01	0.02	<0.01	<0.01
OSM	4.29 ± 0.77	4.18 ± 0.88	3.00 ± 1.40	<0.01	0.02	<0.01	<0.01
SIRT2	2.53 ± 0.92	2.40 ± 0.59	1.33 ± 0.62	<0.01	<0.01	<0.01	<0.01
STAMPB	3.92 ± 0.44	3.83 ± 0.38	3.27 ± 0.41	<0.01	<0.01	<0.01	<0.01
ST1A1	3.90 ± 1.11	3.50 ± 1.06	0.87 ± 0.59	<0.01	<0.01	<0.01	<0.01
TGF-α	4.11 ± 0.45	4.07 ± 0.59	3.56 ± 0.96	0.04	0.18	<0.01	0.04
TGFβ1	7.60 ± 0.22	7.60 ± 0.24	7.32 ± 0.50	0.04	0.16	<0.01	0.01
TNFSF14	6.27 ± 0.50	6.25 ± 0.63	4.35 ± 0.81	<0.01	<0.01	<0.01	<0.01
TWEAK	9.72 ± 0.27	9.78 ± 0.29	9.47 ± 0.51	0.07	0.14	<0.01	<0.01
uPA	9.97 ± 0.42	9.95 ± 0.30	10.19 ± 0.28	0.10	0.20	<0.01	<0.01
VEGFA	10.08 ± 0.66	10.13 ± 0.57	9.53 ± 0.60	0.01	0.08	<0.01	<0.01

Values are mean normalized protein expression-values ± standard deviation. p-values are after false detection rate post hoc correction; unadjusted and adjusted for gender, age, smoking and body mass index. Post hoc power calculation showed very high power > 80%. ASD, atrial septal defect.

Increased levels were observed in 14 biomarkers (19%) comparing patients with an open ASD with controls after adjustment for gender, age, smoking and body mass index. Only one biomarker, Delta and Notch-like epidermal growth factor-related receptor, were decreased in patients with an open ASD compared with controls. We made further adjustment for estimated large ASD in the group of open ASDs. This resulted in minor differences as increased levels were seen in 15 biomarkers (20%).

Comparing patients with a spontaneously closed ASD with controls, we found increased levels in 24 biomarkers (33%) and decreased levels in three biomarkers (4%).

### Pathway overrepresentation analysis

**FIGURE 2:**
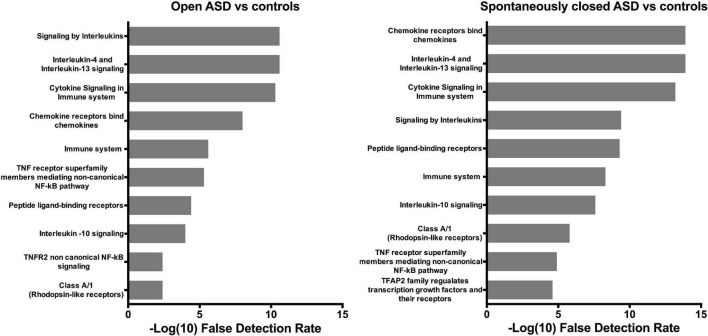
Pathway enrichment analysis. Top 10 pathways significantly enriched in patients with an open atrial septal defect vs. controls (left) and patients with a spontaneously closed atrial septal defect vs. controls (right). Gray bars denote -log (10) false detection rate, which was chosen for graphical purposes. As such, a value > 1.30 corresponds to and false detection rate < 0.05. ASD, atrial septal defect; NF- κB, Nuclear factor kappa-light-chain-enhancer of activated B-cells; TFAP2, Transcription factor activating enhancer binding protein 2; TNF, Tumor necrosis factor; TNFR2, Tumor necrosis factor receptor 2.

The 10 pathways with highest overrepresentation in patients with an open ASD vs. controls and in patients with spontaneously closed ASD vs. controls are depicted in Figure 2. Sirtuin 2 was not identified in the Reactome database, thus the Reactome analysis were made of 13 biomarkers in patients with an open ASD vs. controls and 23 biomarkers in patients with a spontaneously closed ASD vs. controls. Results were similar in both analyses, and the top significantly enriched pathways included Signaling by Interleukins, Interleukin 4, and Interleukin 13 signaling, Cytokine Signaling in Immune System and Chemokine receptors bind Chemokines.

## Discussion

Of 73 detectable inflammatory markers, we found that one out of five were elevated in patients with an open ASD and one out of three biomarkers were elevated in patients with a spontaneously closed defect when comparing with controls. Using an enrichment pathway analysis, we found specific inflammatory pathways to be overrepresented. Thus, patients diagnosed with ASD seem to have a higher inflammatory burden than heart-healthy peers even when the ASD was deemed hemodynamically insignificant or had closed spontaneously in the time between diagnosis in childhood and the present study.

To our knowledge, the present study is the first to investigate a broad array of inflammatory proteins in patients with an ASD. Our results provide novel information about an increased inflammatory activity in patients with ASD. A few studies have evaluated single inflammatory biomarkers in patients with CHD. In patients with simple shunt lesion such as a ventricular septal defect, Zhang et al. have demonstrated changes in acute phase reactants (25). Opotowsky et al. found that 20% of the patients with simple shunt lesions had high-sensitive c-reactive protein levels in the highest quartile, indicating increased inflammatory activity. Furthermore, they found elevated high-sensitive c-reactive protein to be an independent predictor of future cardiovascular events and all-cause mortality (11).

Chamakou et al. found increased Interleukin 6 levels pre-operatively in patients with an ASD admitted for transcatheter closure of the defect compared with healthy controls. Interleukin 6 decreased to control levels 3 months postoperatively, suggesting that closure of the defect may normalize Interleukin 6 levels (26). Remarkably, we found higher levels of 24 inflammatory biomarkers in patients with a spontaneously closed ASD when comparing with controls. It has previously been demonstrated that the majority of defects close within the first 5 years of life (27). The median age of our patients is 24 years, thus presumably the ASD has been spontaneously closed for several years. Still, these patients have increased activity of inflammatory markers. Eleven patients had a hemodynamically significant ASD, and these patients were recommended closure due to right ventricular dilatation or pulmonary artery dilation. A subgroup analysis disclosed no major differences between these supposedly largest defect and the spontaneously closed or small defects. Hemodynamically important ASD are in general of larger size than hemodynamically insignificant defects, indicating that the size of the defect is most likely not related to the degree of inflammation.

In combination with the current literature our data suggest that some of the alterations in the inflammatory responses detected in simple shunt lesions are long lasting and not depending on the continued presence of a shunt. The alterations remain despite removal of the shunt, and they may have long lasting impact.

Inflammatory cells and pathways contribute to the fibrosis in both pulmonary and cardiac tissue and to progression of pathological cardiac remodeling (12, 14). We found higher levels of Vascular endothelial growth factor A, Transforming growth factor beta 1 and Oncostatin M in patients with an ASD compared to healthy controls. These growth factors are known to be involved in endothelial cell proliferation and differentiation, induction of fibrosis, and to be associated with both inflammatory airway diseases and atrial fibrillation (12, 28, 29).

In children with CHD, the increased susceptibility to infectious complications is thought to be due in part to the inflammatory response (9). We have previously demonstrated an increased risk of pneumonia in patients with an ASD (4). The present results suggest that this increased risk could be related to inflammation. The “Interleukin 4 and Interleukin 13 signaling” pathway is enriched in patients with ASD compared with controls. Interleukin 4 and Interleukin 13 are T helper cell type 2 derived cytokines and act as key mediators of tissue fibrosis, especially in pulmonary fibrosis (30) which may also in part explain the previously described lower forced expiratory volume in the first second in this patient group (2).

Patients with an ASD are known to have increased prevalence of atrial fibrillation (3), and we have in this cohort of patients demonstrated an increased burden of asymptomatic arrhythmias including focal atrial tachycardia and non-sustained ventricular arrhythmia (31). Furthermore, patients with ASD have increased risk of stroke (3). Inflammatory pathways not only promote fibrosis and atrial fibrillation, but may also promote a prothrombotic state though endothelial damage and platelet activation in patients with and without atrial fibrillation (15, 32). The Cluster of differentiation 40 has been associated with platelet aggregation and thrombus formation (32, 33). Cluster of differentiation 40 is one of the proteins increased in patients with an ASD in the present study.

Despite being a simple CHD, patients with an ASD have increased mortality compared with background population, and heart failure is the major cause of death (1). Heart failure is associated with circulating inflammatory cytokines, growth factors and monocyte activation (34–36). Chronic inflammation is thought to lead to adverse remodeling and impaired myocardial contractility (36). Our patients are in their twenties to forties and none have heart failure. We speculate that this cohort is too young to demonstrate potential links between inflammation and heart failure.

We used the pathway analysis to receive a more detailed description of the inflammatory biological processes overrepresented in patients with an ASD compared with controls. The majority of the most significant pathways are cytokine-related, as both chemokines and interleukins are defined as cytokines (37). Cytokines are engaged in numerous biological processes, comprising cell growth and differentiation, apoptosis, repair processes, and embryonic development (37, 38). The results of our present study, combined with previous mortality and morbidity findings, makes us speculate whether the inflammatory burden may be present from birth and further studies are needed to, not only confirm our findings, but also provide a deeper understanding. The perspective in understanding this inflammatory response is the potential aid in developing prophylaxis and treatment for complications from CHDs.

### Strengths and limitations

Patients with a spontaneously closed ASD were in average 5 years younger than patients with an open ASD and controls. Low-grade inflammation is found positively correlated with increasing age (39), but we adjusted results to age, and it is thus unlikely that age caused overestimated results in the patients with a spontaneously closed ASD. Furthermore, we adjusted for gender, body mass index, and smoking to limit the risk of confounding, yet there is always a possibility of residual confounding.

In terms of study strengths, the study is based on a nationwide cohort including all diagnosed patients with an ASD in Denmark. Moreover, we have previously shown that there is no difference in age, time of diagnosis and comorbidities between included patients and non-responders in the study (2).

Many patients with a small, unrepaired ASD are asymptomatic, and some are diagnosed by coincidence or in relation to other hospital admittances. Therefore, we can only speculate how many patients are living with an undiagnosed ASD. It would be reasonable to consider, if the patients enrolled in our study, may represent a subgroup of ASD patients with more baseline co-morbidities than the total population of patients with an unrepaired ASD.

## Conclusion

In conclusion, multiple, different inflammatory-related biomarkers and cytokine related pathways are overrepresented in patients with an ASD compared with healthy controls. These findings indicate an altered inflammatory activity, most likely unrelated to the shunt. The increased inflammatory burden may contribute to the pathophysiology of morbidities among patients with an ASD. Our results support that an ASD cannot be considered a simple, benign lesion. Indeed, more studies are needed in order to further characterize the association and any causal relation.

## Data availability statement

The original contributions presented in the study are included in the article/Supplementary material, further inquiries can be directed to the corresponding author/s.

## Ethics statement

The studies involving human participants were reviewed and approved by the Regional Committee on Biomedical Research Ethics of the Central Denmark Region (j.nr. M-2015-197-15). Written informed consent to participate in this study was provided by the participants’ legal guardian/next of kin.

## Author contributions

A-SS, CN, and VH involved in the conceptualization and designing the project, wrote the protocol, and applied for funding and approvals. A-SS, AS, CN, MS, and VH involved in the analysis and interpretation of data. A-SS drafted the manuscript. All authors contributed to the writing and revisions of the manuscript.
