# Supplementary material for: Increased inflammatory markers in adult patients born with an atrial septal defect
Source: Front Cardiovasc Med. 2022 Aug 1;9:925314. doi: 10.3389/fcvm.2022.925314 (PMC9377416; doi:10.3389/fcvm.2022.925314)
Supplement: Supplementary file 1 [file Data_Sheet_1.docx]

**Supplemental materials**

**Online tables 1-3**

| Table 1: Abbreviations and names of all analyzed inflammatory biomarkers | |
| --- | --- |
| Biomarker abbreviation | **Biomarker name** |
| ADA | Adenosine Deaminase |
| ARTN | Artemin |
| AXIN1 | Axin 1 |
| BDNF | Brain-derived neurotrophic factor |
| β-NGF | Beta-nerve growth factor |
| CASP8 | Caspase 8 |
| CCL3 | C-C motif chemokine 3 |
| CCL4 | C-C motif chemokine 4 |
| CCL11 | C-C motif chemokine 11 / Eotaxin |
| CCL19 | C-C motif chemokine 19 |
| CCL20 | C-C motif chemokine 20 |
| CCL23 | C-C motif chemokine 23 |
| CCL25 | C-C motif chemokine 25 |
| CCL28 | C-C motif chemokine 28 |
| CDCP1 | CUB domain-containing protein 1 |
| CD5 | T-cell surface glycoprotein CD5 |
| CD6 | T-cell surface glycoprotein CD6 isoform |
| CD40 | CD40L receptor / TNF-receptor superfamily member 5 |
| CD244 | Natural killer receptor 2B4 |
| CSF1 | Macrophage colony-stimulating factor 1 |
| CST5 | Cystatin D |
| CXCL1 | C-X-C motif chemokine 1 |
| CXCL5 | C-X-C motif chemokine 5 |
| CXCL6 | C-X-C motif chemokine 6 |
| CXCL9 | C-X-C motif chemokine 9 |
| CXCL10 | C-X-C motif chemokine 10 |
| CXCL11 | C-X-C motif chemokine 11 |
| CX3CL1 | Fractalkine |
| DNER | Delta and Notch-like epidermal growth factor-related receptor |
| EBP1 | Eukaryotic translation initiation factor 4E-binding protein 1 |
| ENRAGE | Protein S100-A12 |
| FGF5 | Fibroblast growth factor 5 |
| FGF19 | Fibroblast growth factor 19 |
| FGF21 | Fibroblast growth factor 21 |
| FGF23 | Fibroblast growth factor 23 |
| Flt3L | Fms-related tyrosine kinase 3 ligand |
| GDNF | Glial cell-derived neurotrophic factor |
| HGF | Hepatocyte Growth Factor |
| IFN-γ | Interferon gamma |
| IL1α | Interleukin 1 alpha |
| IL2 | Interleukin 2 |
| IL2Rβ | Interleukin 2 receptor subunit beta |
| IL4 | Interleukin 4 |
| IL5 | Interleukin 5 |
| IL6 | Interleukin 6 |
| IL7 | Interleukin 7 |
| IL8 | Interleukin 8 |
| IL10 | Interleukin 10 |
| IL10Rα | Interleukin 10 receptor subunit alpha |
| IL10Rβ | Interleukin 10 receptor subunit beta |
| IL12β | Interleukin 12 beta |
| IL13 | Interleukin 13 |
| IL15Rα | Interleukin 15 receptor subunit alpha |
| IL17A | Interleukin 17A |
| IL17C | Interleukin 17C |
| IL18 | Interleukin 18 |
| IL18R1 | Interleukin 18 receptor 1 |
| IL20 | Interleukin 20 |
| IL20Rα | Interleukin 20 receptor subunit alpha |
| IL22Rα1 | Interleukin 22 receptor subunit alpha 1 |
| IL24 | Interleukin 24 |
| IL33 | Interleukin 33 |
| LIF | Leukemia inhibitory factor |
| LIFR | Leukemia inhibitory factor receptor |
| MCP1 | Monocyte chemoattractant protein-1 |
| MCP2 | Monocyte chemoattractant protein 2 / CCL8 |
| MCP3 | Monocyte chemoattractant protein-3 |
| MCP4 | monocyte chemoattractant protein 4 |
| MMP1 | Matrix metalloproeinase-1 |
| MMP10 | Matrix metalloproteinase 10 |
| NRTN | Neurturin |
| NT3 | Neurotrophin 3 |
| OPG | Osteoprotegerin |
| OSM | Oncostatin-M |
| PDL1 | Programmed cell death 1 ligand 1 |
| SCF | Stem cell factor |
| SIRT2 | Sirtuin 2 |
| SLAMF | Signaling lymphocytic activation molecule |
| STAMPB | STAM binding protein |
| ST1A1 | Sulfotransferase 1A1 |
| TGFα | Transforming growth factor alpha |
| TGFβ1 | Transforming growth factor beta1 |
| TNF | Tumor necrosis factor |
| TNFβ | Tumor necrosis factor beta |
| TNFRSF9 | Tumor necrosis factor receptor superfamily member 9 |
| TNFSF14 | Tumor necrosis factor ligand superfamily member 14 |
| TRAIL | TNF-related apoptosis-inducing ligand |
| TRANCE | TNF-related activation-induced cytokine |
| TSLP | Thymic stromal lymphopoietin |
| TWEAK | TNF-related weak inducer of apoptosis / TNF ligand superfamily member 12 |
| uPA | Urokinase-type plasminogen activator |
| VEGFA | Vascular endothelial growth factor A |

| Table 2: List of excluded biomarkers | |
| --- | --- |
| Excluded biomarkers, abbreviation (n=19) | Name |
| ARTN | Artemin |
| BDNF | Brain-derived neurotrophic factor |
| FGF5 | Fibroblast growth factor 5 |
| IFN-γ | Interferon gamma |
| IL1α | Interleukin 1 alpha |
| IL2 | Interleukin 2 |
| IL2Rβ | Interleukin 2 receptor subunit beta |
| IL4 | Interleukin 4 |
| IL5 | Interleukin 5 |
| IL13 | Interleukin 13 |
| IL20 | Interleukin 20 |
| IL20Rα | Interleukin 20 receptor subunit alpha |
| IL22Rα1 | Interleukin 22 receptor subunit alpha 1 |
| IL24 | Interleukin 24 |
| IL33 | Interleukin 33 |
| LIF | Leukemia inhibitory factor |
| NRTN | Neurturin |
| TNF | Tumor necrosis factor |
| TSLP | Thymic stromal lymphopoietin |

Inflammatory biomarkers excluded after analysis due to >5% of values below limit of detection.

| Table 3: 44 inflammatory markers with non-significant different levels between ASD patients and controls | | | | | | | |
| --- | --- | --- | --- | --- | --- | --- | --- |
| Biomarker | **Open ASD**  **(n=27)** | **Spont. closed ASD**  **(n=99)** | **Controls**  **(n=23)** | **Open ASD vs controls** | | **Spont. closed ASD vs controls** | |
|  |  |  |  | *Unadjusted*  *p-value* | *Adjusted p-value* | *Unadjusted p-value* | *Adjusted p-value* |
| ADA | 3.71±0.36 | 3.77±0.32 | 3.75±0.30 | 0.72 | 0.75 | 0.91 | 0.75 |
| β-NGF | 1.91±0.28 | 1.91±0.30 | 1.96±0.49 | 0.76 | 0.98 | 0.72 | 0.92 |
| CCL19 | 8.71±0.80 | 8.67±0.64 | 8.46±0.55 | 0.34 | 0.57 | 0.25 | 0.73 |
| CCL20 | 4.65±0.59 | 4.94±0.94 | 4.79±1.11 | 0.72 | 0.75 | 0.64 | 0.91 |
| CCL25 | 6.10±0.55 | 6.02±0.52 | 6.24±0.70 | 0.57 | 1.00 | 0.19 | 0.46 |
| CCL28 | 1.30±0.47 | 1.33±0.52 | 1.40±0.50 | 0.64 | 0.75 | 0.71 | 0.94 |
| CDCP1 | 2.69±0.73 | 2.60±0.58 | 2.64±0.63 | 0.87 | 0.43 | 0.82 | 0.94 |
| CD5 | 5.03±0.29 | 5.03±0.28 | 4.99±0.32 | 0.81 | 0.81 | 0.70 | 0.73 |
| CD6 | 4.58±0.41 | 4.66±0.52 | 4.71±0.56 | 0.50 | 0.63 | 0.79 | 0.73 |
| CD244 | 5.74±0.32 | 5.72±0.31 | 5.65±0.35 | 0.51 | 0.81 | 0.51 | 0.84 |
| CSF1 | 7.91±0.20 | 8.00±0.24 | 7.99±0.27 | 0.41 | 0.34 | 0.87 | 0.91 |
| CST5 | 6.01±0.52 | 5.76±0.47 | 5.82±0.55 | 0.36 | 0.40 | 0.72 | 0.81 |
| CXCL5 | 11.80±0.62 | 11.71±0.72 | 11.07±1.38 | 0.05 | 0.20 | 0.01 | 0.06 |
| CXCL9 | 6.93±0.77 | 6.92±0.68 | 7.12±0.73 | 0.52 | 0.91 | 0.35 | 0.52 |
| CXCL10 | 7.67±0.99 | 7.75±0.97 | 7.32±0.55 | 0.25 | 0.29 | 0.10 | 0.11 |
| CX3CL1 | 5.62±0.32 | 5.64±0.36 | 5.85±0.41 | 0.09 | 0.23 | 0.05 | 0.12 |
| EBP1 | 7.34±0.65 | 7.32±0.59 | 6.89±0.74 | 0.07 | 0.37 | 0.01 | 0.08 |
| ENRAGE | 4.92±0.64 | 4.89±0.66 | 4.64±0.75 | 0.30 | 0.91 | 0.23 | 0.55 |
| FGF19 | 7.74±0.81 | 7.77±0.83 | 8.09±0.99 | 0.32 | 0.17 | 0.23 | 0.52 |
| FGF21 | 4.64±1.05 | 4.83±1.30 | 4.59±1.37 | 0.91 | 0.73 | 0.58 | 0.70 |
| FGF23 | 1.28±0.30 | 1.23±0.34 | 1.32±0.64 | 0.84 | 0.81 | 0.48 | 0.91 |
| Flt3L | 8.58±0.50 | 8.65±0.39 | 8.85±0.53 | 0.14 | 0.08 | 0.11 | 0.40 |
| IL6 | 2.84±0.94 | 2.66±0.76 | 2.55±0.50 | 0.33 | 0.94 | 0.65 | 0.81 |
| IL7 | 4.59±0.43 | 4.70±0.52 | 4.78±0.94 | 0.50 | 0.58 | 0.71 | 0.73 |
| IL8 | 6.11±0.45 | 6.00±0.53 | 6.08±0.76 | 0.91 | 0.91 | 0.70 | 0.60 |
| IL10 | 2.85±0.56 | 2.99±0.61 | 2.91±0.42 | 0.77 | 0.71 | 0.68 | 0.81 |
| IL10Rα | 1.38±0.55 | 1.46±0.65 | 1.32±0.63 | 0.79 | 0.37 | 0.50 | 0.77 |
| IL10Rβ | 6.66±0.28 | 6.68±0.25 | 6.76±0.31 | 0.36 | 0.40 | 0.34 | 0.20 |
| IL15Rα | 0.51±0.27 | 0.40±0.19 | 0.33±0.17 | 0.03 | 0.08 | 0.17 | 0.22 |
| IL17A | 0.62±0.46 | 0.63±0.55 | 0.58±0.66 | 0.84 | 0.77 | 0.79 | 0.91 |
| IL17C | 1.41±0.52 | 1.35±0.53 | 1.39±0.42 | 0.91 | 0.91 | 0.81 | 0.96 |
| IL18 | 7.54±0.48 | 7.48±0.53 | 7.36±0.38 | 0.29 | 0.47 | 0.48 | 0.92 |
| IL18R1 | 6.73±0.49 | 6.95±0.38 | 6.84±0.30 | 0.51 | 0.07 | 0.30 | 0.88 |
| LIFR | 2.92±0.13 | 2.97±0.24 | 3.07±0.33 | 0.07 | 0.11 | 0.17 | 0.70 |
| MMP10 | 5.72±0.98 | 5.74±0.62 | 5.87±0.51 | 0.67 | 0.29 | 0.51 | 0.60 |
| NT3 | 1.45±0.32 | 1.40±0.41 | 1.39±0.33 | 0.67 | 0.79 | 0.96 | 0.94 |
| OPG | 10.17±0.38 | 10.19±0.36 | 10.35±0.49 | 0.25 | 0.29 | 0.14 | 0.23 |
| PDL1 | 3.67±0.37 | 3.79±0.58 | 3.55 ±0.27 | 0.34 | 0.80 | 0.12 | 0.40 |
| SCF | 9.72±0.32 | 9.56±0.37 | 9.66±0.39 | 0.72 | 0.60 | 0.36 | 0.73 |
| SLAMF | 1.98±0.98 | 1.73±0.46 | 1.73±0.63 | 0.44 | 0.60 | 0.97 | 0.81 |
| TNFβ | 4.24±0.59 | 4.18±0.39 | 3.97±0.51 | 0.19 | 0.08 | 0.08 | 0.18 |
| TNFRSF9 | 5.89±0.48 | 5.87±0.34 | 5.88±0.38 | 0.94 | 0.85 | 0.91 | 0.83 |
| TRAIL | 8.57±0.33 | 8.61±0.34 | 8.60±0.19 | 0.79 | 0.29 | 0.94 | 0.92 |
| TRANCE | 4.42±0.59 | 4.48±0.62 | 4.20±0.54 | 0.31 | 0.29 | 0.11 | 0.25 |

Values are mean NPX-value±standard deviation. p-values are after false discovery rate post-hoc correction; unadjusted and adjusted for gender, age, smoking and body mass index.
